# *Chelidonium majus* Induces Apoptosis of Human Ovarian Cancer Cells via ATF3-Mediated Regulation of Foxo3a by Tip60

**DOI:** 10.4014/jmb.2109.09030

**Published:** 2022-02-16

**Authors:** Lei Shen, Soon Lee, Jong Cheon Joo, Eunmi Hong, Zhen Yang Cui, Eunbi Jo, Soo Jung Park, Hyun-Jin Jang

**Affiliations:** 1 Aerospace Center Hospital, Beijing 100049, P.R. China; 2 Division of Analytical Science, Korea Basic Science Institute, Daejeon 34133, Republic of Korea; 3 Division of Analytical Science, University of Science and Technology, Daejeon 34113, Republic of Korea; 4 Department of Sasang Constitutional Medicine, College of Korean Medicine, Wonkwang University, Iksan 54538, Republic of Korea; 5 Rehabilitation Medicine College, Weifang Medical University, Weifang 261042, P.R. China; 6 Department of Life Science and Research Institute for Natural Sciences, College of Natural Sciences, Hanyang University, Seoul 04763, Republic of Korea; 7 Department of Sasang Constitutional Medicine, College of Korean Medicine, Woosuk University, Jeonju 54987, Republic of Korea; 8 Laboratory of Chemical Biology and Genomics, Korea Research Institute of Bioscience and Biotechnology, Daejeon 34141, Republic of Korea

**Keywords:** *Chelidonium majus*, ovarian cancer, apoptosis, SKOV3, ATF3, Tip60

## Abstract

Forkhead transcription factor 3a (Foxo3a) is believed to be a tumor suppressor as its inactivation leads to cell transformation and tumor development. However, further investigation is required regarding the involvement of the activating transcription factor 3 (ATF3)-mediated Tat-interactive protein 60 (Tip60)/Foxo3a pathway in cancer cell apoptosis. This study demonstrated that *Chelidonium majus* upregulated the expression of ATF3 and Tip60 and promoted Foxo3a nuclear translocation, ultimately increasing the level of Bcl-2-associated X protein (Bax) protein. ATF3 overexpression stimulated Tip60 expression, while ATF3 inhibition by siRNA repressed Tip60 expression. Furthermore, siRNA-mediated Tip60 inhibition significantly promoted Foxo3a phosphorylation, leading to blockade of Foxo3a translocation into the nucleus. Thus, we were able to deduce that ATF3 mediates the regulation of Foxo3a by Tip60. Moreover, siRNA-mediated Foxo3a inhibition suppressed the expression of Bax and subsequent apoptosis. Taken together, our data demonstrate that *Chelidonium majus* induces SKOV-3 cell death by increasing ATF3 levels and its downstream proteins Tip60 and Foxo3a. This suggests a potential therapeutic role of *Chelidonium majus* against ovarian cancer.

## Introduction

*Chelidonium majus* L., one of the most important medicinal plants in the Papaveraceae family, is widely used in European countries and in traditional Chinese medicine [1]. Traditionally, *C. majus* is used to treat liver disorders, gastrointestinal problems and stomach cramps in Europe and Western Asia, and cancer, peptic ulcer, jaundice and toothache in Eastern Asia [2, 3]. Originally introduced in the Jiu Huang Ben Cao, an ancient Chinese medical book, *C. majus* is mentioned in the Chinese pharmacopoeia for its therapeutical effects on several diseases such as whooping cough, gastritis and spasms [4]. Jonathan Hartwell recorded in his compendium ‘Plants used against cancer’ that *C. majus* has been used in monastic medicine for a long time for the treatment of various diseases [5].

The anti-tumor activity of *C. majus* extract has been identified in previous studies. The European Medicines Agency addressed the anti-tumor effect of *C. majus*, along with its anti-spasmodic and anti-inflammatory activities [6]. A strong cytotoxic activity against pancreas (PANC-1) and colon (HT-29) tumor cell lines, and a moderate cytotoxic activity against breast (MDA-MB-231) tumor cell lines, have also been reported [7]. *C. majus* had a growth inhibitory effect on both human epidermoid carcinoma A431 cells and NSCLC cells, including H1975 and PC9 [8].

The major constituents of *C. majus* are isoquinoline alkaloids (namely chelidonine, protopine, chelerythrine, sanguinarine, berberine and coptisine), flavonoids and phenolic acids [9]. Its crude extracts and purified compounds are involved in a wide range of biological activities, exhibiting anti-inflammatory, anti-microbial, immunomodulatory, anti-tumor, choleretic, hepatoprotective and analgesic effects [10-14]. Alkaloids in *C. majus* have been studied for years and have already been determined as substances with anti-tumor potential [14, 15]. Previous research has suggested that the anti-tumor effects of *C. majus* extract are likely due to chelidonine, chelerythrine, sanguinarine and berberine.

Chelidonine is a major alkaloid component in *C. majus*, and its therapeutic effects on various health disorders and diseases have been studied. It consists of isoquinoline alkaloids, notably protoberberine and benzophenanthridine alkaloids, and has been shown to exhibit anti-cancer effects and induce cancer cell death in the early disease stages [16]. Furthermore, chelidonine has anti-migratory and anti-invasive effects on various cancer cell types [6, 17, 18] and may affect the cell cycle checkpoints and MAPK signaling pathways within cells [19]. It has been described that chelidonine alone shows more potent effects than *C. majus* [17, 20].

Ovarian cancer is one of the three most common gynecological malignant tumors [21] and the seventh most common cancer in women worldwide [22]. Its 5-year survival rate is only 30%, and over two-thirds of ovarian cancer patients are at stage III or IV when diagnosed [23]. The current standard treatment is chemotherapy, which applies platinum drugs, such as carboplatin and taxanes [24].

Activating transcription factor 3 (ATF3) is a member of the ATF/cAMP-response element-binding protein family of transcription factors and it contains the basic region-leucine zipper DNA binding domain [25, 26]. It is rapidly upregulated following exposure to hepatotoxic chemicals or DNA-damaging agents and UV/ionizing radiation [27, 28]. Previous research reported that ATF3 has pro-apoptotic roles in ovarian cancer cells [29] and suppresses prostate cancer with phosphatase and tensin homolog dysfunction [30]. In MCF10CA1a human breast cancer cells, ATF3 overexpression attenuates apoptosis and enhances motility when the cells are malignant [29], while facilitating apoptosis in untransformed cells [31]. Tip60, which contains MOZ, Ybf2/Sas3, SAS2, and Tip60 (MYST) domains, is a member of the MYST family of histone acetyltransferases and was initially identified as a 60-kDa human immunodeficiency virus Tat-interacting protein. It is involved in a variety of cellular processes (apoptosis, mammary tumorigenesis, and transcriptional regulation) [32, 33], and has been associated with cancer cell proliferation in breast cancer, colorectal cancer, and cutaneous T-cell lymphoma [34-36]. However, whether Tip60 is a tumor-suppressor gene or a proto-oncogene is unclear [37].

Mammalian forkhead transcription factors (Foxo) belong to the O ('other') class of the Fox superfamily [38]. Among Foxo1, Foxo3, Foxo4 and Foxo6, which have been recognized for their engagement in cellular proliferation, function and demise [39], Foxo3a is especially crucial in oncogenesis and suppressing the growth of various human cancers [40], and its subcellular localization, distribution, and phosphorylation are often closely linked to colon [41], prostate [42], bladder [43] and breast [44] cancer prognosis.

In this study, we investigated how *C. majus* engages in ovarian cancer cell apoptosis by activating the ATF3-Tip60 pathway. Our results demonstrate the process by which *C. majus*-mediated ATF3 induces Tip60 and promotes Foxo3a nuclear translocation, leading to apoptosis in human ovarian cancer cells via the caspase-3-dependent pathway.

## Materials and Methods

### Preparation of *C. majus* Extract

*C. majus* was provided by Wonkwang University Jeonju Korean Medicine Hospital (Jeollabuk-do, Republic of Korea). Fresh bodies or mycelia of *C. majus* were placed in 99.9% methyl alcohol at 45°C for 2 h, and then filtered (pore size: 1 μm) before concentration. Water was used as the solvent for the total extract obtained after drying (200 g, yield (w/w), 11%). Part of this sample (number. 027-073) was stored at the Korea Research Institute of Bioscience and Biotechnology Biological Resource Center (http://biorp.kribb.re.kr), and collected from Odoosan, Myoshan-myeon, Hapcheon-gun, Gyeongsangnam-do, Republic of Korea.

### Cell Culture and Cell Viability Assay

SKOV-3, OVCAR-3 and MDAH-2774 cell lines (American Type Culture Collection, USA) were cultured in Dulbecco’s Modified Eagle’s Medium (DMEM) supplemented with 10% (v/v) fetal bovine serum and 1% (w/v) antibiotic-antimycotic, and maintained in 5% (v/v) CO_2_ at 37°C. To observe changes in cell viability, we seeded cells into 96-well plates (around 5 × 10^3^ cells/well), 24 h prior to *C. majus* exposure. After 24 and 48 h of treatment, the cells were maintained in media containing DojinDo Cell Counting Kit-8 solution (Japan) for 2 h at 37°C. The absorbance was measured at 450 nm and we determined the half maximal inhibitory concentration (IC_50_) with the triplicate data of *C. majus*-induced cytotoxicity.

### Detection of Apoptosis by Propidium Iodide/Annexin V Staining

An Annexin V-FITC Apoptosis Detection Kit (Sigma, USA) was utilized to observe the apoptosis of SKOV-3 cells in response to *C. majus*. After 24 or 48 h of treatment, the cells were washed with PBS and exposed to Annexin V binding buffer, Annexin V-FITC conjugate (0.1 μg/ml) and PI (2 μg/ml). Following incubation at room temperature for 10 min protected from light, the fluorescence of Annexin V (530/30 nm filter) and PI (670 nm filter) in the samples were evaluated at 488 nm excitation with the Guava flow cytometry system (Millipore, USA).

### Transmission Electron Microscopy

Samples for transmission electron microscopy were prepared by fixing the *C. majus*-treated SKOV-3 cells on ice for 2 h with glutaraldehyde (2.5%) and osmium tetroxide (1%). After washing with PBS, dehydration, and embedding with Epon 812 mixture, the blocks polymerized for 24 h at 70°C were sectioned into ultrathin layers, which were stained with uranyl acetate, rinsed, and then stained with lead citrate. Samples were examined with the JEM-1400Plus Bio-HVEM and JEM-1000BEF systems (Jeol, Japan).

### Microarray Analysis

With a 44K cDNA chip, we performed transcription profiling of RNA obtained from SKOV-3 cells exposed to vehicle or *C. majus* (500 μg/ml). RNA reverse transcription with amino allyl dUTP was conducted to synthesize cDNA probes, which were labeled with Cy3 or Cy5 dyes. Genes with the global M and log2 (R/G) values exceeding |1.0| (two-fold) with *p* < 0.05 were considered to be differentially expressed. The significance of the expression changes was assessed by Student’s t-test. Finally, the genes were classified into specific categories to determine the biological significance of the changes.

### Gene Ontology-Based Network Analysis

The genes and proteins identified were screened through Ingenuity pathway analysis (IPA; http://www.ingenuity.com) according to their ontology-related networks, such as apoptotic signaling pathways. We aimed to evaluate how the identified genes function biologically by optimizing the generation of closely connected networks based on the available expression profiles.

### Cloning of Transcription Factor Binding Sequences into Vectors

pGreenFireTM-Pathway Reporter Constructs with a minimal CMV promoter were digested using EcoRI and SpeI restriction enzymes (NEB, USA). We amplified the sequences encoding the transcription factor binding sites of interest by PCR and separated them via gel extraction. This was followed by ligation of the purified DNA fragments using T4 ligase and transformation by heat shock. The plasmid sequences were verified to confirm the constructs that allowed us to monitor specific transcription factors through green fluorescence protein (GFP) and luciferase (SBI System Biosciences, USA) expression.

### Lentivirus Packaging

Using Hillymax (Dojindo, Japan), human embryonic kidney (HEK) 293T cells were transfected with the cloned expression vector and lentivirus packaging vectors. Following 5 h of incubation, the vector-containing media were replaced with media containing 5% FBS, without antibiotics. The virus was acquired approximately 48 h later, passed through a 0.4 μm syringe filter, and maintained at -80°C until use.

### Transcription Factor Network Analysis

The expression of the transcription factors was analyzed as described in our previous study [45]. SKOV-3 cells cultured on a black 96-well plate were transduced by lentivirus for 24 h. Cells that were successfully transduced underwent selection by 7 days of exposure to puromycin. After treatment with *C. majus* (200 μg/ml) for 48 h, we measured the fluorescence of the cells every 12 h with a TriStar² S LB 942 plate reader (Berthold Technologies, Bad Wildbad, Germany). The excitation and detection wavelengths were 480 nm and 510 nm, respectively. The activities of the transcription factors were determined based on the data normalized to the non-treated group. The data were submitted through BTNET (http://ibtnet.korea.ac.kr/) to generate the schematics of the transcription factor activation and the relevant regulatory networks, as shown previously [46].

### Fractionation and Protein Extraction

SKOV-3 cells exposed to *C. majus* extract for 48 h were added to homogenization buffer A (protease inhibitor cocktail, 25 mM Tris (pH 7.5), 1 mM DTT, 0.5 mM EGTA, 1 mM PMSF, 2 mM EDTA and 0.02% Triton X-100). After homogenization and centrifugation (100,000 ×*g*, 30 min), the pellet was sonicated in a suspension of homogenization Buffer A including 1% of Triton X-100, followed by 30 min of incubation at 4°C with gentle rocking. Then, the supernatant nuclear fraction of the centrifuged product was collected for western blotting.

### Plasmid Construction and RNA Interference

We purchased the plasmid construction of ATF3 from GeneScript (USA) and used pcDNA3.1 (Invitrogen, USA) to assemble the expression vectors. The small interfering RNAs (siRNAs) had the following nucleotide sequences: ATF3 siRNA - 5’-CGA UUU GGA GGU ACC AUA AAG GAU U-3’; Tip60 siRNA - 5'-AAG AAC GGA AGU GUG AUA UGU-3' (ST PHARM, Korea). Foxo3a siRNA was purchased from Cell Signaling (USA). SKOV3 cells were transfected with siRNA using Lipofectamine RNAiMAX reagent (Invitrogen) according to the manufacturer’s instructions.

### Western Blotting

Total cell lysates were obtained from cells homogenized in 20 mM Tris-HCl including protease inhibitor (Roche, Switzerland), left on ice for 30 min, and centrifuged for at 15,000 ×*g* and 4°C for 10 min. The protein concentration of each sample was determined according to the bicinchoninic acid assay. SDS-polyacrylamide gels were used to separate the denatured proteins, which were then transferred onto nitrocellulose membranes (0.2 μm). The membranes were blocked with skimmed milk (5% (w/v), dissolved in Tris-buffered saline with Tween-20 (TTBS)) for 1 h, and the antibodies against the following proteins were added: ATF, Tip60, Bax, Bcl2, β-actin (purchased from Santa Cruz (USA)), Caspase3, Foxo3a, and p-Foxo3a (acquired from Cell Signaling). The membranes were washed (thrice, 5 min each) with TTBS (0.1% (v/v)), exposed to goat anti-mouse or rabbit anti-goat IgG (1:2000 dilution) at room temperature for 1 h, then washed again (thrice, 5 min each) with TTBS. Bands were visualized via a ChemiDoc MP system (Bio-Rad, USA). Band densities were determined through ImageJ (National Institutes of Health, USA), and β-actin was used as the normalization standard for quantification.

### 3D Spheroid Culture

For 3D spheroid culture, SKOV-3 cells were seeded into 96-well, ultra-low attachment microplates (1000 cells/well) and centrifuged at 200 ×*g* for 5 min. The cells were incubated for 3 days for spheroid formation, followed by *C. majus* treatment at the indicated concentrations. Cell culture media were replaced with fresh *C. majus*-containing media 3 days after the initial treatment. Changes in the morphology of the spheroids were observed for a week and the diameters of the spheres were measured.

### Statistical Analyses

All statistical analyses were conducted with GraphPad Prism version 5 (GraphPad, USA). Student’s *t*-test was performed to assess the significance of the differences between groups. The half-maximal inhibitory concentration (IC_50_) value of the treatments was deduced based on its nonlinear curve containing five data points with mean ± SD.

## Results

### *C. majus* Extract Inhibits Ovarian Cancer Cell Growth

As chelidonine has been studied numerously in regard to its anti-cancer effects, we validated the presence of chelidonine in the *C. majus* extract. Using HPLC analysis, the sample (10 μl) was monitored at 290 nm with a UV detector (Table S1). The chelidonine content in the *C. majus* extract was found to be 20.1 mg/g (Fig. S1).

**Fig. 1:**
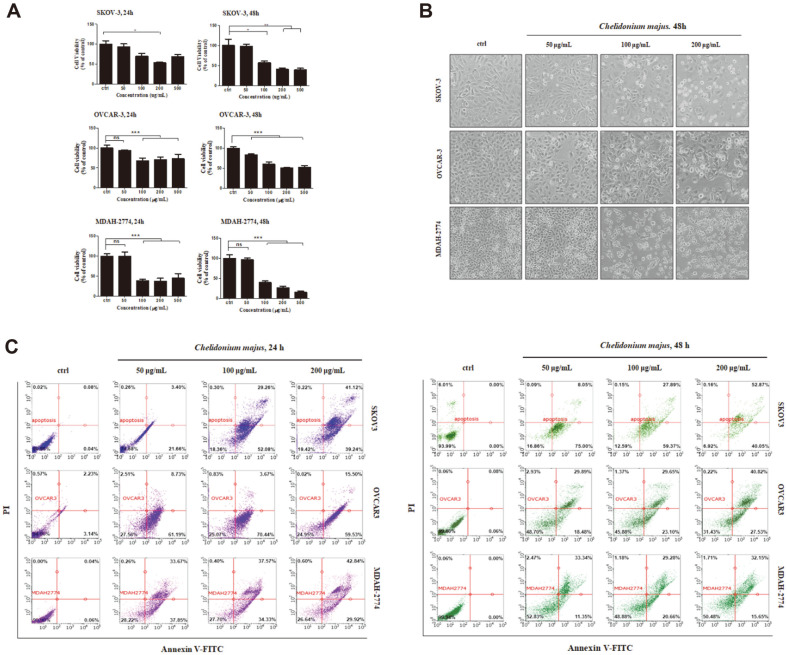
*C. majus* extract induces apoptosis in SKOV-3, OVCAR-3 and MDAH-2773 cells. A. *C. majus* inhibits ovarian cancer cell growth. Cells were treated with 0, 50, 100, 200 and 500 μg/ml of *C. majus* extract for 24 and 48 h. B. Changes in the morphology of SKOV-3, OVCAR03 and MDAH-2774 cells after *C. majus* treatment for 24 and 48 h. X400 magnification. C. FACS analysis showing apoptosis in ovarian cancer cells after *C. majus* treatment. Apoptosis was analyzed by flow cytometry using Annexin V and PI staining. Data represent the mean ± SD of three independent experiments. **p < 0.01 and ***p < 0.001 versus vehicle-treated cells.

To examine how *C. majus* inhibits the growth of ovarian cancer cells, we exposed SKOV-3, OVCAR-3 and MDAH-2774 cells to 0, 100, 200, 300, 400, or 500 μg/ml of the extract. Fig. 1A shows that after 48 h of incubation, all three cell lines showed a dose-dependent suppression of proliferation. The IC_50_ value of *C. majus* at 24 h was determined to be 200 μg/ml, as it inhibited around 50% of growth of the three cell lines (Fig. 1A).

We also examined the effect of *C. majus* treatment on human mesenchymal stem cells (hMSC) to assess its toxicity on normal cells (Fig. S2). The results showed that unlike the ovarian cancer cell lines, their cell viability remained above 60%, even when treated up to a concentration of 500 μg/ml for 24 and 48 h.

The visualization of cell death following *C. majus* treatment was carried out by light microscopy. Drastic changes in cell morphology after 48 h of exposure to 200 μg/ml *C. majus* are presented in Fig. 1B. We noticed that many cells had shrunk and were detaching from the dish surface, or becoming buoyant, as a sign of apoptosis. In comparison, 50 μg/ml *C. majus* treatment for 24 h induced fewer changes in morphology.

### *C. majus* Extract Induces Apoptosis in Ovarian Cancer Cells

Flow cytometry was performed to observe the apoptotic activities of SKOV-3, OVCAR-3 and MDAH-2774 cells labeled with Annexin V and PI after exposure to 0, 50, 100, or 200 μg/ml of *C. majus*. The cell populations in early apoptosis (lower right quadrant) and late apoptosis or necrosis (upper quadrant) are represented in dot plots. When we treated cells with 50 μg/ml *C. majus* for 24 h, the fraction of Annexin V-stained viable cells was remarkably reduced in OVCAR-3 (95% to 27%) and MDAH-2774 (99% to 28%) cells (Fig. 1C). SKOV-3 cells exhibited a relatively smaller reduction in their viable cell population (99% to 75%) within 24 h, but after 48 h showed a larger decrease (94% to 17%). Thus, *C. majus* induced apoptosis of OVCAR-3 and MDAH-2774 cells after 24 h, whereas 48 h of treatment was required to induce SKOV-3 cell apoptosis.

### *C. majus*-Treated Ovarian Cancer Cells Exhibit Apoptotic Bodies

We chose to carry out further experiments on SKOV-3, which is known as one of the most invasive ovarian cancer cells lines. Originally isolated from the ascitic fluid of a patient, SKOV-3 cells are closely linked to the late stages of the disease [47]. Therefore, by investigating the response of SKOV-3 cells in particular, we attempted to confirm the effect of *C. majus* on ovarian cancer, including the more aggressive types of cells.

**Fig. 2:**
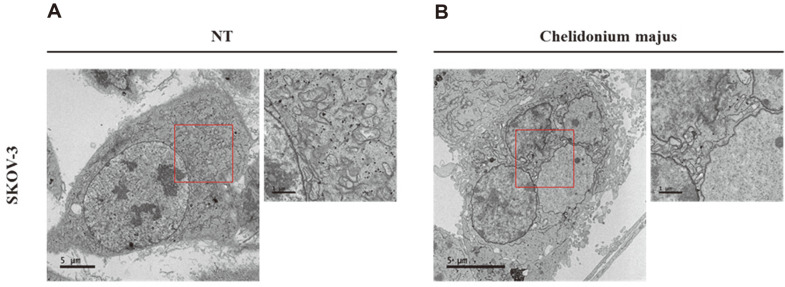
Morphological ultrastructural appearance of ovarian cancer cells after *C. majus* treatment using transmission electron microscopy. **A**. Untreated SKOV-3 cells and B. SKOV-3 cells treated with 500 μg/ml *C. majus* for 24 h were observed. In *C. majus*-treated cells, apoptotic bodies with spherical shapes were observed detaching from the cell surface. These contained lumps of chromatin that were fragmented and segregated. Representative images are shown and a scale bar is marked under each image.

Using transmission electron microscopy (TEM), we were able to confirm apoptosis following *C. majus* treatment and gained a deeper understanding of the changes in the SKOV-3 cell ultrastructure while it was undergoing apoptosis (Fig. 2). After 500 μg/ml of treatment, we noticed apoptotic bodies, which appeared as spherical shapes detaching from the cell surface. These contained lumps of chromatin that were fragmented and segregated. The structure of the mitochondria and Golgi apparatus were also disrupted by *C. majus* treatment (Fig. 2B). This was quite unlike the untreated SKOV-3 cells, which maintained a normal nuclear architecture, undamaged plasma membranes, and chromatin folding (Fig. 2A).

### *C. majus* Alters the Gene Expression of Ovarian Cancer Cells

By running a microarray analysis (with an Agilent Human GE 8×60K Microarray) of SKOV-3 cells after exposure to 500 μg/ml *C. majus*, we obtained a list of genes that may be associated with its anti-cancer effects. We evaluated 63,242 unique genes, and found 356 and 180 genes up- and downregulated, respectively, after 48 h of *C. majus* treatment. The gene ontology (GO) enrichment analysis assessed these genes based on the Database for Annotation, Visualization and Integrated Discovery (http://david.abcc.ncifcrf.gov/).

**Fig. 3:**
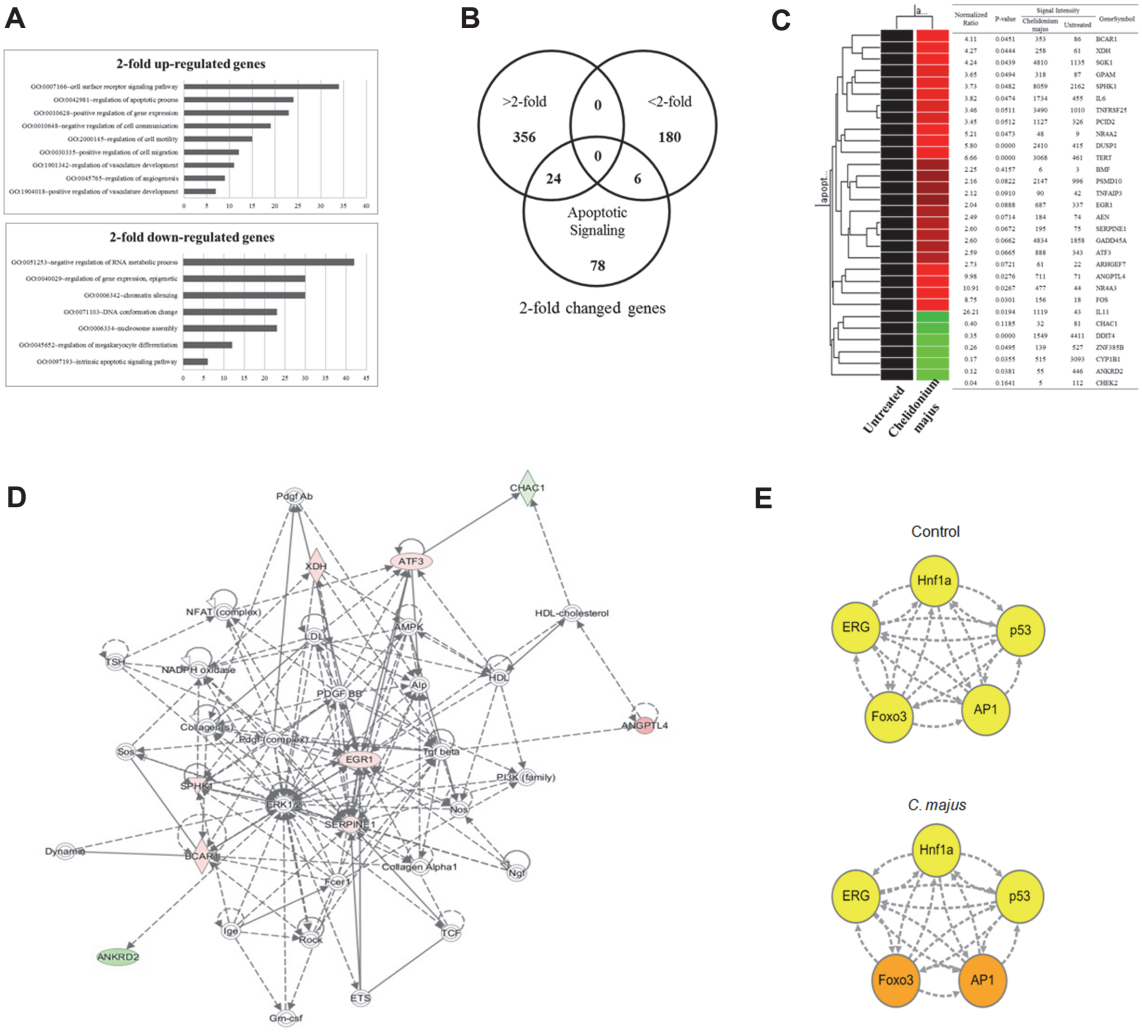
*C. majus* upregulates apoptosis-associated genes. **A**. Gene ontology analysis of genes with expressional differences between *C. majus*-treated (500 μg/ml) and untreated SKOV-3 cells. **B**. A Venn diagram showing the number of genes that were regulated (>two-fold, <two-fold, and apoptosis-associated genes). **C**. A network of apoptotic genes regulated by *C. majus*. Genes involved in the signaling network of *C. majus*-treated SKOV-3 cells were colored using IPA. Nodes in red represent the upregulated genes, and green the downregulated genes. **D**. Apoptotic genes affected after *C. majus* treatment shown in hierarchical clustering. Red circles represent upregulation and the green circles represent downregulation of the corresponding transcriptional factor. Arrows with dotted lines represent inferences and those with solid lines indicate prior knowledge. E. Changes in the transcriptional network of SKOV-3 cells after *C. majus* treatment.

Fig. 3B presents the number of genes that showed a change of greater than two-fold in the GO analysis. Most of the upregulated genes were associated with cell surface receptor signaling, apoptosis regulation, cell motility, cell migration, and vasculature development regulation (Fig. 3A). The downregulated genes were involved in RNA metabolism, chromatin silencing, DNA conformational changes, nucleosome assembly, and intrinsic apoptosis signaling (Fig. 3A). Next, to sort out potential regulators of apoptosis, differences in the gene expression between *C. majus*-treated cells and the control were noted based on the GeneCards database (http://www.genecards.org/) (Figs. 3C and 3D). The names and their signaling networks of apoptosis-related genes are given in Fig. 3D.

Furthermore, to determine the regulation of transcriptional factors involved, a transcription network analysis was conducted. As shown in Fig. 3E, C. majus specifically induced Foxo3 and activator protein-1 (AP-1) transcriptional activity. Hepatocyte nuclear factor-1 alpha (Hnf1a), p53 and erythroblast transformation-specific-related gene (ERG) were not affected.

Thus, we were able to pinpoint that ATF3 and Foxo3a had major roles in the interactome network of SKOV-3 cells that led to apoptosis in response to *C. majus* treatment.

### *C. majus* Increases ATF3 and Tip60 Expression and Inhibits Foxo3a Phosphorylation, Which Induces Foxo3a Nuclear Translocation

**Fig. 4:**
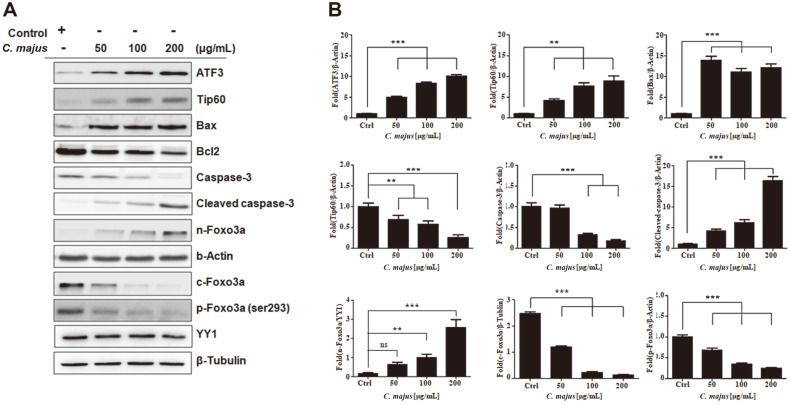
*C. majus* promotes the activation of Foxo3a pathways by regulating ATF3/Tat-interactive protein 60 signaling. **A**. Representative western blot of SKOV-3 cells, depicting the changes in the protein levels of ATF3, Tip60, caspase- 3, Bax and Foxo3a in response to *C. majus* treatment. **B**. The density of the bands after normalization against β-actin. Data are the mean ± SEM values from three independent experiments.

Western blot analysis confirmed that the expression of ATF3 and Tip60 increased after 48 h of *C. majus* treatment (50, 100, and 200 μg/ml) (Fig. 4). Given that Foxo3a has been shown to have tumor-suppressing functions, we examined changes in Foxo3a expression. We found that p-Foxo3a expression markedly declined after 48 h of treatment (Fig. 4).

Subsequently, we investigated whether the de-phosphorylation of Foxo3a led to Foxo3a nuclear translocation. The nuclear and cytosolic proteins were extracted and the expression of Foxo3a in each fraction was quantified by western blotting (Fig. 4). Nuclear Foxo3a levels were increased according to dose, while the cytosolic Foxo3a levels were decreased significantly. The pro-apoptotic proteins downstream of Foxo3a, including cleaved caspase-3 and Bax, were upregulated, while the anti-apoptotic B-cell lymphoma-2 (Bcl-2) expression decreased in a dose-dependent manner.

Expression changes after *C. majus* treatment were also examined in the other two ovarian cancer cell lines, OVCAR-3 and MDAH-2774 (Fig. S3). Similar to SKOV-3 cells, p-Foxo3a levels decreased and ATF3 levels increased in response to 48 h of *C. majus* treatment. Tip60 expression levels showed an increase in MDAH-2774 cells and a tendency to decrease in OVCAR-3 cells. Cleaved caspase-3 levels increased in both cell lines, as a sign of apoptosis.

### ATF3 Phosphorylates Foxo3a and Regulates Foxo3a Nuclear Translocation through the ATF3-Mediated Tip60 Signaling Pathway

**Fig. 5:**
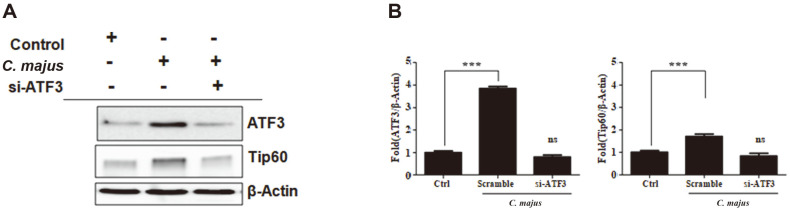
*C. majus* promotes the activation of Tat-interactive protein 60 by regulating ATF3 signaling. **A**. Representative western blot of SKOV-3 cells after exposure to *C. majus*. *C. majus* increased ATF3 and Tip60 expression. **B**. The density of the bands after normalization against β-actin. Data are mean ± SEM values from three independent experiments.

**Fig. 6:**
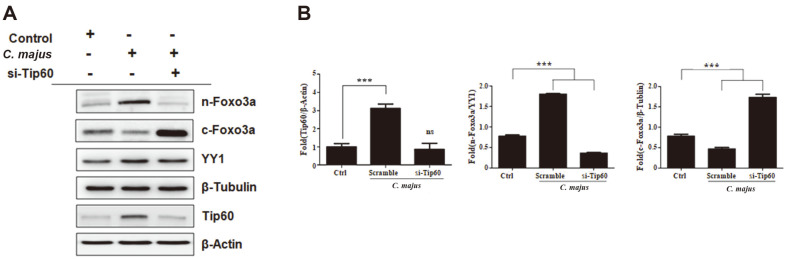
*C. majus* promotes the activation of Foxo3a by regulating Tip60 signaling. **A**. Representative western blot of SKOV-3 cells treated with control or Tip60-targeting siRNA. *C. majus* increased Tip60 expression and Foxo3a nuclear translocation in SKOV-3 cells, but this effect was abolished in Tip60-targeted SKOV-3 cells. **B**. The density of the bands after normalization against β-actin. Data are the mean ± SEM values from three independent experiments.

To assess the role of ATF3 in facilitating Tip60 activation, we induced ATF3 silencing using siRNA. When exposed to *C. majus*, the ATF3-silenced SKOV-3 cells showed suppressed ATF3 and Tip60 protein expression (Fig. 5), unlike normal SKOV-3 cells that presented upregulated levels of ATF3 and Tip60. Subsequently, to determine the involvement of Tip60 in Foxo3a regulation, Tip60 was silenced using siRNA. Exposure to *C. majus* increased Tip60 expression in normal SKOV-3 cells, but this effect was attenuated when the cells were exposed to Tip60-targeting siRNA (Fig. 6). Tip60-silenced cells also showed significantly suppressed levels of nuclear Foxo3a and upregulated cytoplasmic Foxo3a after *C. majus* treatment, suggesting that Tip60 is essential for the translocation of Foxo3a into the nucleus (Fig. 6).

### Foxo3a Nuclear Translocation Triggered by *C. majus* Facilitates Bax Expression

**Fig. 7:**
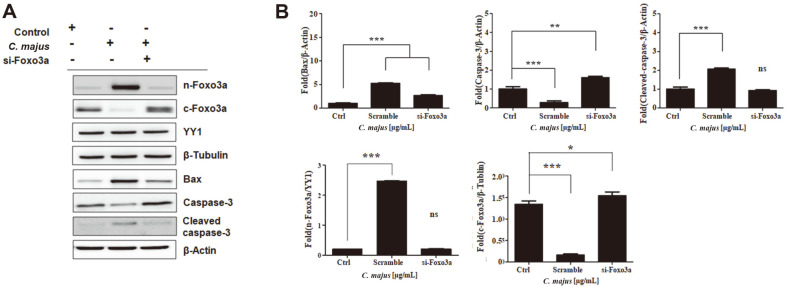
Foxo3a regulates *C. majus*–induced caspase-3 activation in SKOV-3 cells. **A**. Representative western blot of SKOV-3 cells treated with control or Foxo3a-targeting siRNA. The protein levels of Foxo3a, caspase-3 and Bax protein are shown. **B**. The density of the bands after normalization against β-actin. Data are mean ± SEM values from three independent experiments.

To confirm that *C. majus* induces apoptosis of SKOV-3 cells through Foxo3a, we examined the levels of apoptotic proteins after silencing Foxo3a. Foxo3a nuclear translocation and the expression of cleaved caspase-3 and Bax were determined (Fig. 7). Silencing of Foxo3a by siRNA led to attenuated levels of cleaved caspase-3 and Bax, indicating that these pro-apoptotic proteins are upregulated by increased nuclear Foxo3a levels.

### *C. majus* Inhibits the Growth of SKOV-3 Spheroid in 3D Culture

**Fig. 8:**
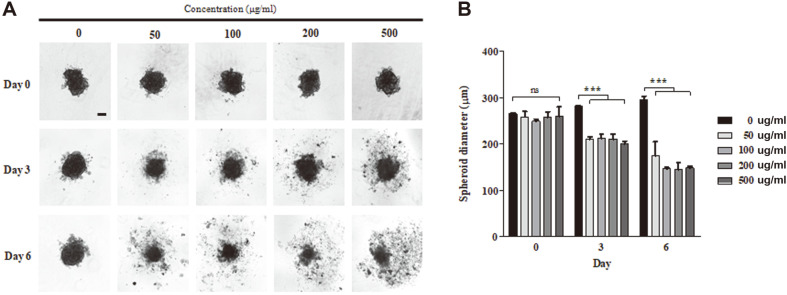
*C. majus* inhibits the growth of SKOV-3 spheroids in 3D culture. Cells were cultured for 3 days post-seeding to allow spheroid formation before drug treatment. **A**. Representative microscopic images of spheroids on day 0, 3, and 6 of treatment. Scale bar represents 100 μm. **B**. Diameter analysis of spheroids on day 0, 3 and 6 of treatment. Data represent the mean ± SD of three independent experiments. ***p < 0.0001 vs. vehicle cells

For further verification of the effect of *C. majus* in an environment with a better representation of the in vivo tissue, we generated 3D spheroids of SKOV-3 cells. After 3 days post-seeding, the cell aggregates became compact, forming spheroids (Day 0) (Fig. 8). Over the one week of treatment, spheroids exposed to *C. majus* showed impeded growth and compactness characterized by disassembled cells and a reduction in the size of the spheroid core. In contrast, the spheroids treated with vehicle maintained their compactness and presented a more spherical feature.

## Discussion

*C. majus* has been reported to possess various biological functions, facilitating anti-inflammatory, antimicrobial, and antioxidant effects [48]. It is also known for its roles regarding cell death, cell proliferation, migration, and invasiveness, in liver, bladder, pulmonary, gastrointestinal, and prostate cancer [17, 18, 49]. Such anti-tumor activities are mainly achieved by regulating biologically important signaling pathways leading to apoptosis. However, further research is required on its effects on the apoptotic pathways regarding ATF3 and Foxo3a.

In the present study, we demonstrated that the ATF3/Tip60/Foxo3a pathway triggers apoptosis in SKOV-3 cells after *C. majus* treatment. Our results showed that ATF3-mediated Tip60 upregulation and p-Foxo3a downregulation preceded SKOV-3 cancer cell apoptosis, suggesting that *C. majus*-induced SKOV-3 cell apoptosis was mediated by Foxo3a phosphorylation and dephosphorylation. The repressed p-Foxo3a levels facilitated Foxo3a nuclear translocation, leading to increased levels of apoptosis-facilitating proteins, such as cleaved caspase-3 and Bax. *C. majus* enhanced n-Foxo3a levels by activating Tip60 through ATF3. Tip60 suppression by siRNA facilitated Foxo3a phosphorylation, subsequently downregulating n-Foxo3a, Bax, and cleaved caspase-3 levels. Finally, blocking Foxo3a downregulated *C. majus*-induced Bax expression.

Many studies have suggested that ATF3 has an oncogenic role, although others have described ATF as an inhibitor of tumorigenesis [28]. This implies that the physiological function of ATF3 may vary among the different types of cancer. ATF3 protects malignant human breast cancer cells from apoptosis and promotes their metastatic potential, whereas its overexpression promotes apoptosis of PC3 human prostate cancer cells [18, 50]. We found that ATF3 and Tip60 expression increased after *C. majus* treatment in a dose-dependent manner (Fig. 4). Foxo proteins induce cell death signaling by increasing the expression of multiple pro-apoptotic Bcl-2 proteins, stimulating the expression of death receptor ligands such as Fas ligand and tumor necrosis factor-related apoptosis-inducing ligands, or upregulating the levels of various cyclin-dependent kinase inhibitors [51].

Phosphorylation of Foxo3a induces its nuclear export. Once exported from the nucleus, p-Foxo3a may then be ubiquitylated and undergo degradation [52]. Activated Foxo3a upregulates Bax and induces cell apoptosis through the expression of genes necessary for cell death [53]. Therefore, the data presented in this study indicate that *C. majus* treatment triggers SKOV-3 cell apoptosis by Tip60 activation through ATF3. Although the role of Foxo3a in suppressing tumor growth has not been reported, we assessed the changes in Foxo3a expression after *C. majus* exposure. Total Foxo3a expression was unaffected, while p-Foxo3a, which is the inactive form, was downregulated in a dose-dependent fashion (Fig. 3C). This suggests that Foxo3a was activated and translocated into the nucleus, as decreased p-Foxo3a levels upregulate n-Foxo3a [54].

Foxo3a suppression by siRNA-mediated Tip60 inhibition significantly suppressed *C. majus*–induced cancer cell death. We also observed that ATF3 overexpression facilitated Tip60 expression, while ATF3 suppression by siRNA downregulated Tip60 levels in response to *C. majus* treatment (Fig. 5). From these results, we were able to establish that ATF3 was responsible for Foxo3a dephosphorylation, which consequently leads to SKOV-3 cell apoptosis.

It remains unclear how Foxo3a regulates apoptosis in a protein kinase B-dependent manner after *C. majus* treatment. Phosphatase activities regulated by Foxo3a may be linked to Foxo3a dephosphorylation. Our results show that Foxo3a increased the level of Bax, a pro-apoptotic gene, which was followed by caspase triggering the cascade of apoptosis. Mitochondrial apoptosis has been reported to be highly dependent on Bax levels upregulated by Foxo3a and the mitochondrial translocation that follows [55]. Therefore, we further examined the effects of inhibited Foxo3a expression on Bax and cleaved caspase-3 levels, which are associated with SKOV-3 apoptosis. When Foxo3a expression was suppressed by siRNA, Bax levels were downregulated (Fig. 7), which led us to believe that SKOV-3 cell apoptosis following *C. majus* treatment would be attenuated by Foxo3a inhibition. Our findings are in line with previously reported data that demonstrate the effects of *C. majus* on other types of cancer cells [56, 57].

The expression changes of the key proteins of the ATF3/Foxo3a pathway showed a similar tendency, across all three cell lines. We observed increased levels of ATF3 and p-Foxo3a, and subsequent upregulation of the pro-apoptotic cleaved-caspase 3 protein. The downregulation of Tip60 observed in the OVCAR-3 cell line suggests that Foxo3a nuclear translocation in this particular cell line may be modulated by factors other than Tip60. It may be related to the R248Q mutation of p53 in OVCAR-3 cells, which the other two cell lines do not carry [58]. The role of Tip60 in the p53-induced pathway of apoptosis has been reported in previous studies [59]. Additionally, low Tip60 levels are correlated with p53 mutations, indicating that Tip60 may function as a tumor suppressor in some types of cancers [32].

It is worth noting that *C. majus* showed greater inhibitory effects on 2D cell proliferation compared to 3D spheroid formation. Stronger drug resistance is a phenomenon often observed in 3D culture models of cancer [60, 61], which form multi-layers as opposed to mono-layered 2D culture. This may be due to many factors, such as differences in cell density, the expression of drug resistance genes, and subsequently, the diffusion capacity of the drugs [61]. Therefore, although somewhat less evident in 3D spheroids, we believe that the inhibitory effects of *C. majus* suggest it has potential as a therapeutic for ovarian cancer patients.

In this study, we showed that *C. majus* induces apoptosis of SKOV-3 cancer cells through Foxo3a signaling mediated by ATF3 and Tip60. Treatment of *C. majus* led to Foxo3a nuclear translocation and p-Foxo3a downregulation, and subsequent expression of apoptotic proteins. Further examination using 3D culture demonstrated that *C. majus* has growth inhibitory effects on ovarian cancer cell spheroids. Our results suggest that *C. majus* may be beneficial for the treatment of ovarian cancer patients in the future.

## Supplemental Materials

Supplementary data for this paper are available on-line only at http://jmb.or.kr.
