# Supplementary material for: Chelidonium majus Induces Apoptosis of Human Ovarian Cancer Cells via ATF3-Mediated Regulation of Foxo3a by Tip60
Source: J Microbiol Biotechnol. 2022 Feb 16;32(4):493–503. doi: 10.4014/jmb.2109.09030 (PMC9628819; doi:10.4014/jmb.2109.09030)
Supplement: Supplementary file 1 [file jmb-32-4-493-supple.pdf]

J. Microbiol. Biotechnol.
https://doi.org/10.4014/jmb.2109.09030

Supplemental Materials

Chelidonium majus induces apoptosis in human ovarian cancer cell via ATF3-mediated regulation of Foxo3a by Tip60

Lei Shen [1†], Soon Lee [2,3†], Jong Cheon Joo [4†], Eunmi Hong [2], Zhen Yang Cui [5], Eunbi Jo [6], Soo Jung Park [7*] and Hyun-Jin Jang [8*]

[1]Aerospace Center Hospital, Beijing 100049, China

[2]Division of Analytical Science, Korea Basic Science Institute, Daejeon 34133, Republic of Korea

[3]Division of Analytical Science, University of Science and Technology, Daejeon 34113, Republic of Korea

[4]Department of Sasang Constitutional Medicine, College of Korean Medicine, Wonkwang University, Iksan, Republic of Korea.

[5]Rehabilitation Medicine College, Weifang Medical University, Weifang 261042, China

[6]Department of Life Science and Research Institute for Natural Sciences, College of Natural Sciences, Hanyang University, Seoul 04763, Republic of Korea

[7]Department of Sasang Constitutional Medicine, College of Korean Medicine, Woosuk University, Jeonju 54987, Republic of Korea

[8]Laboratory of Chemical Biology and Genomics, Korea Research Institute of Bioscience and Biotechnology, Daejeon 34141, Republic of Korea

Table S1

Table S1. Condition of high performance liquid chromatography analysis of *Chelidonium majus* extract components

Detector	UV 290 nm
Column	Sunfire C18, 4.6X250 mm
Column temperature	40 °C
Mobile phase	Acetonitrile: 1% phosphoric acid (18:82)
Flow rate	1.2 ml/min

Figure S1

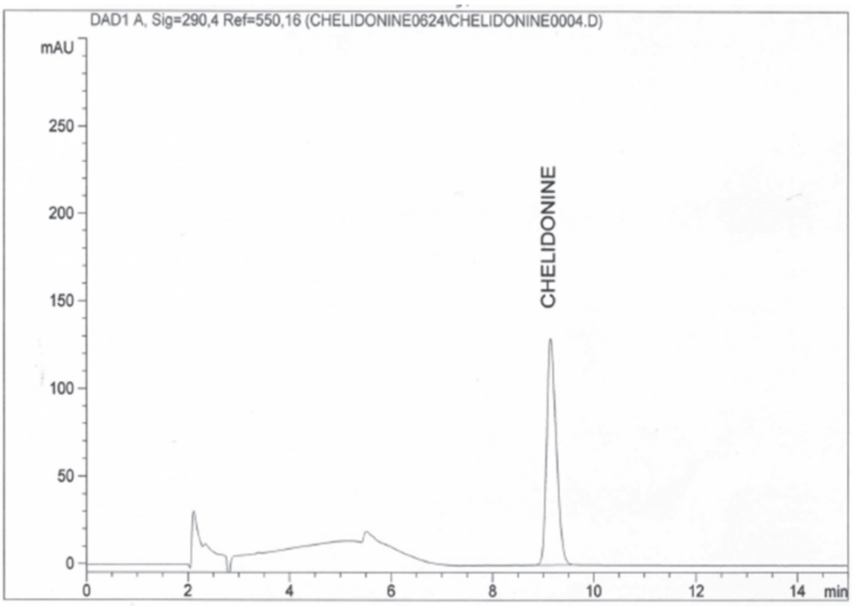

Standard

RetTime(min)=9.151, Area(mAU*s)=1692.71326

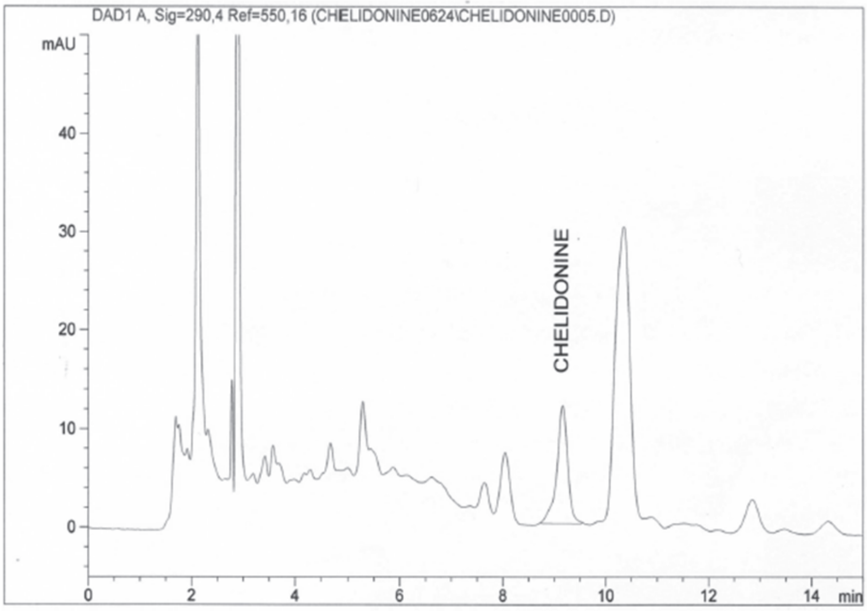

Chelidonium majus extract

RetTime(min)=9.167, Area(mAU*s)=175.76923

Figure S1. Quantitative analysis of chelidonine in *Chelidonium majus* extract via high performance liquid chromatography (HPLC) (unit: mg/g).

Figure S2

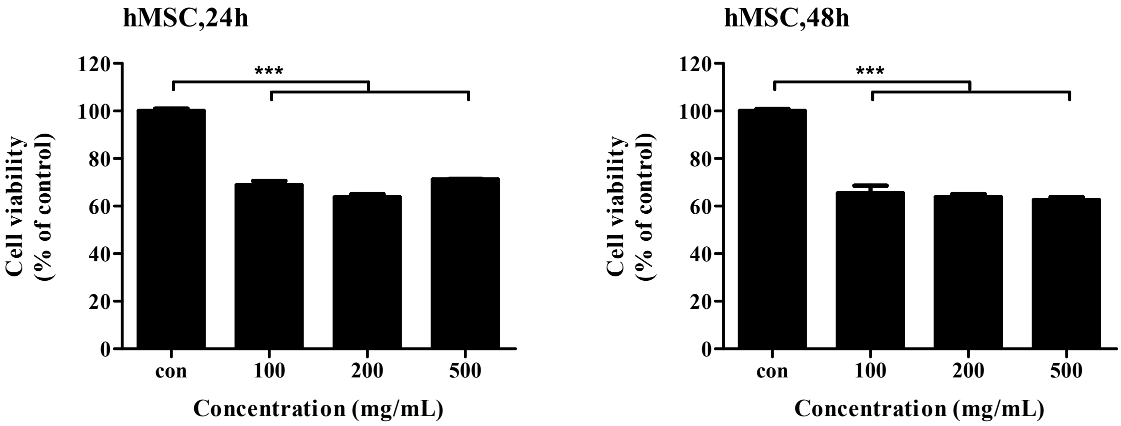

Figure S1. Cell cytotoxicity of Chelidonium majus on human mesenchymal stem cells (hMSC). Cells were treated with 0, 50, 100, 200 and 500 µg/mL of Chelidonium majus extract for 24 and 48 h.

Figure S3

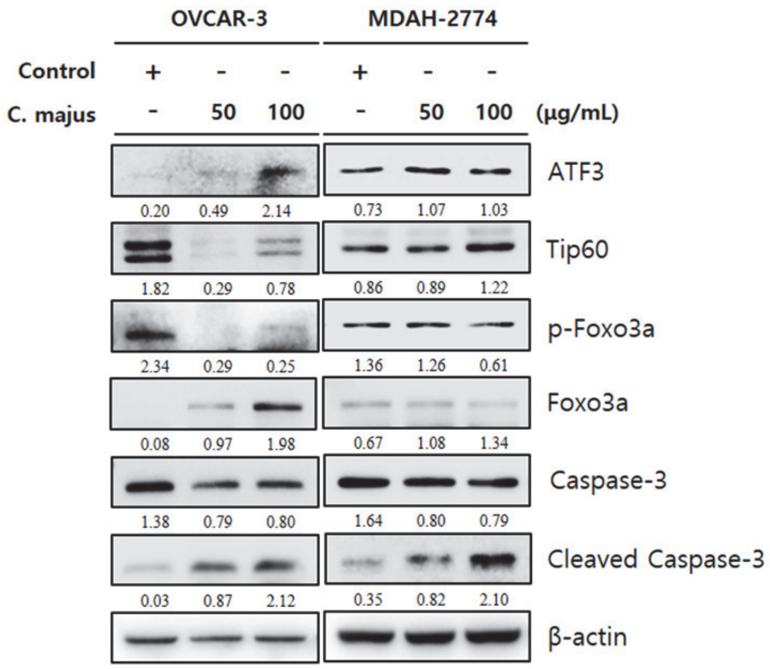

Figure S2. *Chelidonium majus* promotes the activation of forkhead transcription factor 3a pathways by regulating activating transcription factor 3/Tat-interactive protein 60 signaling. Representative western blot of OVCAR-3 and MDAH-2774 cells. The changes in the protein levels of activating transcription factor (ATF3), Tat-interactive protein 60 (Tip60), caspase-3, Bcl-2-associated X protein (Bax) and forkhead transcription factor 3a (Foxo3a) in response to *Chelidonium majus* treatment are shown. Numbers under the bands represent the density normalized against β-actin.
